# Q-BioLiP: A Comprehensive Resource for Quaternary Structure-based Protein–ligand Interactions

**DOI:** 10.1093/gpbjnl/qzae001

**Published:** 2024-01-04

**Authors:** Hong Wei, Wenkai Wang, Zhenling Peng, Jianyi Yang

**Affiliations:** School of Mathematical Sciences, Nankai University, Tianjin 300071, China; School of Mathematical Sciences, Nankai University, Tianjin 300071, China; MOE Frontiers Science Center for Nonlinear Expectations, Research Center for Mathematics and Interdisciplinary Sciences, Shandong University, Qingdao 266237, China; MOE Frontiers Science Center for Nonlinear Expectations, Research Center for Mathematics and Interdisciplinary Sciences, Shandong University, Qingdao 266237, China

**Keywords:** Protein–ligand interaction, Quaternary structure, Protein–ligand binding site, Protein–protein interaction, Binding affinity

## Abstract

Since its establishment in 2013, BioLiP has become one of the widely used resources for protein–ligand interactions. Nevertheless, several known issues occurred with it over the past decade. For example, the protein–ligand interactions are represented in the form of single chain-based tertiary structures, which may be inappropriate as many interactions involve multiple protein chains (known as quaternary structures). We sought to address these issues, resulting in Q-BioLiP, a comprehensive resource for quaternary structure-based protein–ligand interactions. The major features of Q-BioLiP include: (1) representing protein structures in the form of quaternary structures rather than single chain-based tertiary structures; (2) pairing DNA/RNA chains properly rather than separation; (3) providing both experimental and predicted binding affinities; (4) retaining both biologically relevant and irrelevant interactions to alleviate the wrong justification of ligands’ biological relevance; and (5) developing a new quaternary structure-based algorithm for the modelling of protein–ligand complex structure. With these new features, Q-BioLiP is expected to be a valuable resource for studying biomolecule interactions, including protein–small molecule interaction, protein–metal ion interaction, protein–peptide interaction, protein–protein interaction, protein–DNA/RNA interaction, and RNA–small molecule interaction. Q-BioLiP is freely available at https://yanglab.qd.sdu.edu.cn/Q-BioLiP/.

## Introduction

The biological functions of many proteins are achieved by interacting with other biomolecules, which are referred to as ligands. A collection of high-quality data for protein–ligand interactions is essential to enable related computational studies [1–5], such as in the prediction of protein–ligand binding sites [6–11], the prediction of binding affinity [12,13], and protein–ligand docking [14–16]. BioLiP is a database that collects 3-dimensional (3D) structures of biologically relevant protein–ligand interactions [17]. It has emerged as one of the most widely used resources for investigating protein–ligand interactions.

There are several known inherent issues with the data in BioLiP. First, the protein structures in BioLiP are represented in single chain-based tertiary structures. Nonetheless, the functional form of many proteins is in quaternary structures, typically comprising multiple interacting chains. Consequently, some vital protein–ligand interactions are not captured in the BioLiP data due to the incompleteness of the protein structure [18], particularly when a ligand simultaneously interacts with multiple protein chains. For example, the hemoglobin protein can only transport oxygen in the form of a tetramer, which is composed of four chains. Second, DNA ligands in BioLiP are presented in a single-chain format, which is inconsistent with the fact that DNA typically forms a double-helix structure consisting of two complementary chains. The third issue is the potential misjudgement of the biological relevance. BioLiP employs an empirical rule to determine whether a protein–ligand interaction is biologically relevant or not. Consequently, a protein–ligand interaction that is deemed biologically irrelevant is excluded from BioLiP, resulting in the problem of missing data, when a biologically relevant interaction is mistakenly judged as irrelevant.

In this study, we introduce an enhanced version of BioLiP, named Q-BioLiP, to address the aforementioned issues. The major updates include the representation of protein structures in quaternary structures, the retention of both biologically relevant and irrelevant interactions, the paired-chain structures for DNA/RNA ligands, the adoption of macromolecular Crystallographic Information File (mmCIF) format [19] rather than Protein Data Bank (PDB) format [20] to deal with huge molecules that exceed the capability of the PDB format, and the inclusion of computed binding affinity. Last but not least, we provide an efficient template-based approach to ligand-binding site prediction using the Q-BioLiP data, which allows for the structure input consisting of either single chain or multiple chains.

## Database construction

### Overview of Q-BioLiP

**Figure 1:**
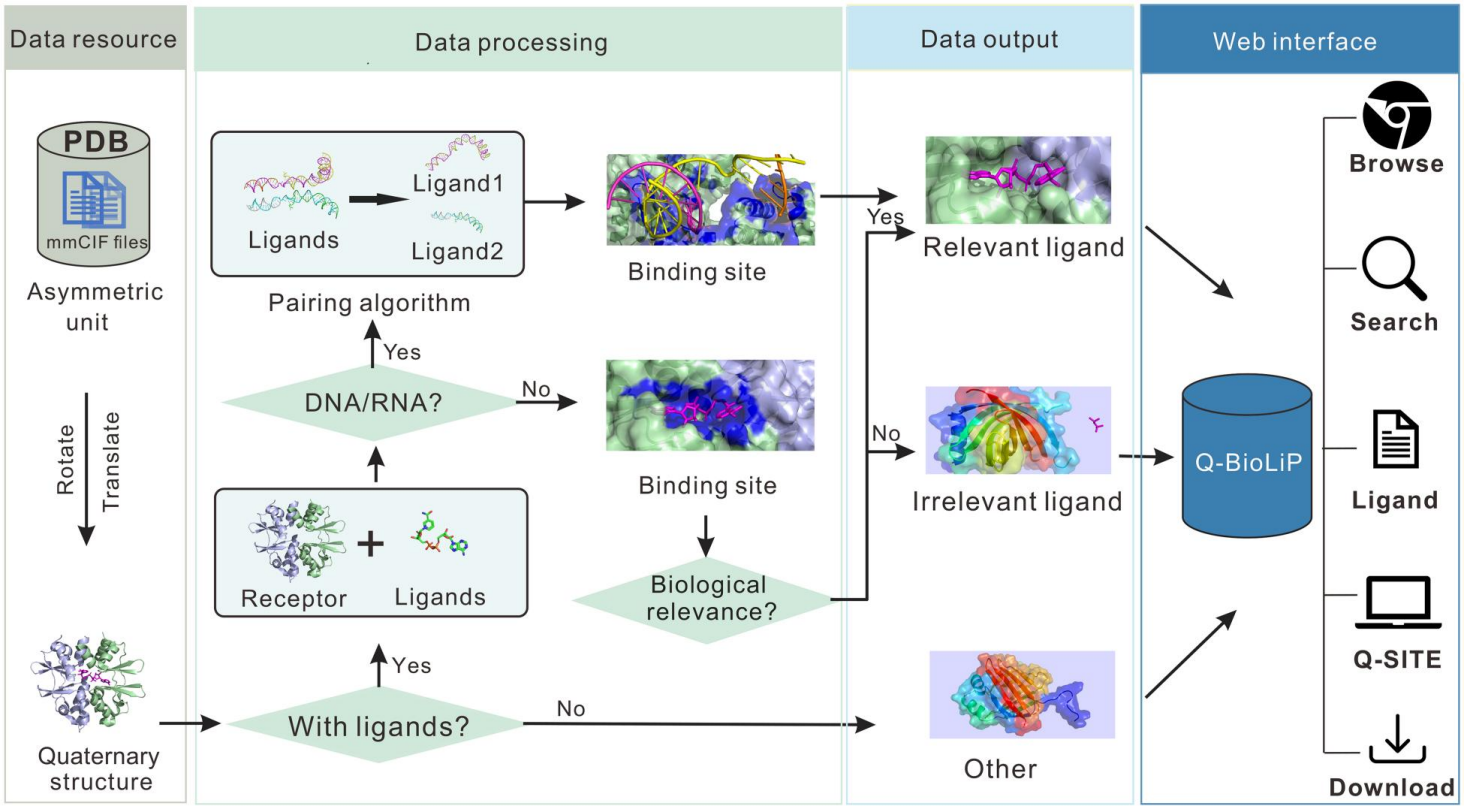
Workflow of Q-BioLiP Quaternary structures were first generated from the asymmetric unit files in PDB. The quaternary structures were then processed into three categories: biologically relevant interactions, biologically irrelevant interactions, and structures with ligands or proteins only. All the data are accessible through a web interface. mmCIF, macromolecular Crystallographic Information File; PDB, Protein Data Bank.

The flowchart for building Q-BioLiP is presented in **Figure 1**. First, starting from the asymmetric unit files (in mmCIF format), quaternary structures (known as biological units) were generated using the rotation and translation matrices stored in the mmCIF files. Then, receptors and ligands were extracted from the quaternary structures. In this work, receptors are defined as proteins consisting of ≥ 30 amino acids (AAs) in each polypeptide chain, while other molecules are defined as ligands (including small molecules, peptides consisting of < 30 AAs, and DNA/RNA). As base pairing can be formed between DNA/RNA chains, a heuristic algorithm (see “An effective DNA/RNA pairing algorithm”) was proposed to pair the DNA/RNA chains. As done in BioLiP [17], the biological relevance of each protein–ligand complex was assessed with a semi-manual procedure. For the sake of completeness, Q-BioLiP also retains quaternary structures with ligands or receptors only. Thus, the data deposited in Q-BioLiP fall into three categories: biologically relevant protein–ligand interactions, biologically irrelevant protein–ligand interactions, and structures with ligands or proteins only. They can be accessed through a user-friendly web interface at https://yanglab.qd.sdu.edu.cn/Q-BioLiP/.

### Procedure for database construction

The raw data were downloaded from the PDB database [20]. The procedure for the construction of Q-BioLiP consists of three major steps.

#### Generation of quaternary structures

For each PDB entry, the asymmetric unit (in mmCIF format) was first downloaded. One or more quaternary structures were generated according to the rotation matrices and the translation vectors stored in the mmCIF file’s records, *i.e.*, “pdbx_struct_assembly”, “pdbx_struct_assembly_gen”, and “pdbx_struct_oper_list”. Modified residues were converted to standard ones based on the record “pdbx_struct_mod_residue”. For nuclear magnetic resonance (NMR) structures, only the first model was taken into consideration in the procedure.

#### Extraction of receptor and ligands from quaternary structures

Each quaternary structure was separated into one receptor and multiple ligands (when they exist). The receptor consists of protein chains with ≥ 30 AAs, while ligands can be small molecules, DNA/RNA, or peptides with < 30 AAs.

#### Annotations of protein–ligand interactions

The interaction between the receptor and each ligand was annotated in terms of binding residues, biological relevance, binding affinity, and area of binding interface.

##### Binding residues

Binding residues were determined based on the atomic distances between the protein and ligand atoms. A residue was considered as a binding residue if its closest atomic distance to the ligand is less than a defined threshold, which is the sum of the Van der Waals radius of the corresponding atoms plus 0.5 Å [21].

##### Biological relevance

Small molecules that are utilized to help determine the structure of proteins but do not have any biological activity were considered biologically irrelevant. Peptides and nucleic acids were regarded as biologically relevant to receptors without the need for assessment. We adopted a similar semi-manual procedure used in BioLiP to assess the biological relevance of small molecules. Briefly, a list of 465 small molecules that are commonly used in protein structure determination was collected manually (available at the Download page). Small molecules outside this list were regarded as biologically relevant by default. For molecules in this list, a hierarchical procedure was employed, which involves computation of the number of binding residues, text mining of the PubMed abstract, and manual verification of suspicious entries. As a result of utilizing quaternary structure in this context, two of the criteria used in BioLiP, namely the ligand occurrence number and the continuity of binding residues, were deactivated. More details can be found in BioLiP [17].

##### Binding affinity

The strength of protein–ligand interaction is commonly referred to as binding affinity. Experimental ligand-binding affinities were collected from three databases: Binding MOAD [2], PDBbind [3], and BindingDB [4]. However, only a limited portion (9.8%) of PDB entries have experimental binding affinity data. To address this issue, predicted binding affinity data were provided in Q-BioLiP. The predicted binding affinity data were obtained based on a few well-established physics-based tools, including X-Score [22], ITScore [14], and AutoDock Vina [16]. To generate a consensus binding affinity prediction, a regression model was trained to fit the real binding affinity by taking the three scores as input.

##### Area of binding interface

Besides binding affinity, the area of the binding interface is provided in Q-BioLiP. Motivated by DOCKGROUND [23], we measured the binding interface area *S* as below:

(1) $$S=\frac{1}{2}\left(S_{\text{prot}}\;+\;S_{\text{lig}}\;-\;S_{\text{com}}\right)$$

where *S*_prot_, *S*_lig_, and *S*_com_ are the solvent-accessible surface areas calculated by the program FreeSASA [24], with the protein, ligand, and ligand–protein complex structures as inputs, respectively.

### An effective DNA/RNA pairing algorithm

As is well known, DNA typically forms a double-helix structure with two complementary chains. Inter-chain interactions are also witnessed between different DNA and RNA chains. However, in BioLiP, DNA/RNA is simply divided into separated chains, making the DNA/RNA structures incomplete. Thus, it is necessary to correctly pair DNA/RNA chains to keep them intact.

To address the aforementioned issue, we proposed a simple yet effective pairing algorithm for DNA/RNA chains. To pair the DNA/RNA chains, we first parsed the secondary structure of the whole DNA/RNA structure using the Dissecting the Spatial Structure of RNA (DSSR) program [25]. Then, the pairing state of each nucleotide was derived from the output of DSSR. Two chains were paired together if there are at least three inter-chain base pairs. Furthermore, in the case of a chain consisting of one or two nucleotides, the pairing was preserved as long as all of its nucleotides are paired with another chain.

We have validated the algorithm using a randomly selected set of 100 structures (list is available at https://yanglab.qd.sdu.edu.cn/Q-BioLiP/DATA/pair_100.txt). The ground truth was obtained through manual inspection. The algorithm described above produced pairings that are identical to the ground truth, indicating its effectiveness. Nevertheless, incorrect pairings may also occur for RNAs with new patterns. To deal with such issues, we will keep improving our algorithm.

## Data content and discussion

### Comparison with BioLiP

The major differences between the data in Q-BioLiP and BioLiP are summarized in **Table 1**, including the following five aspects.

**Table 1:** The major differences between the data in Q-BioLiP and BioLiP

Feature	Q-BioLiP	BioLiP
Source data format	mmCIF	PDB
Protein structure	Quaternary structure	Tertiary structure
State of DNA/RNA chains	Paired chains	Separated chains
Binding affinity	Experimental + predicted	Experimental only
Data category	All PDB structures	Biologically relevant interactions only

*Note*: mmCIF, macromolecular Crystallographic Information File; PDB, Protein Data Bank.

#### Source data format

Due to the limitation of the PDB format, structures with more than 62 chains such as many cryo-electron microscopy (cryo-EM) structures are ignored in BioLiP. The source data processed by Q-BioLiP are in mmCIF rather than PDB format, which solves the issue of missing structures in BioLiP.

#### Protein structure

**Figure 2:**
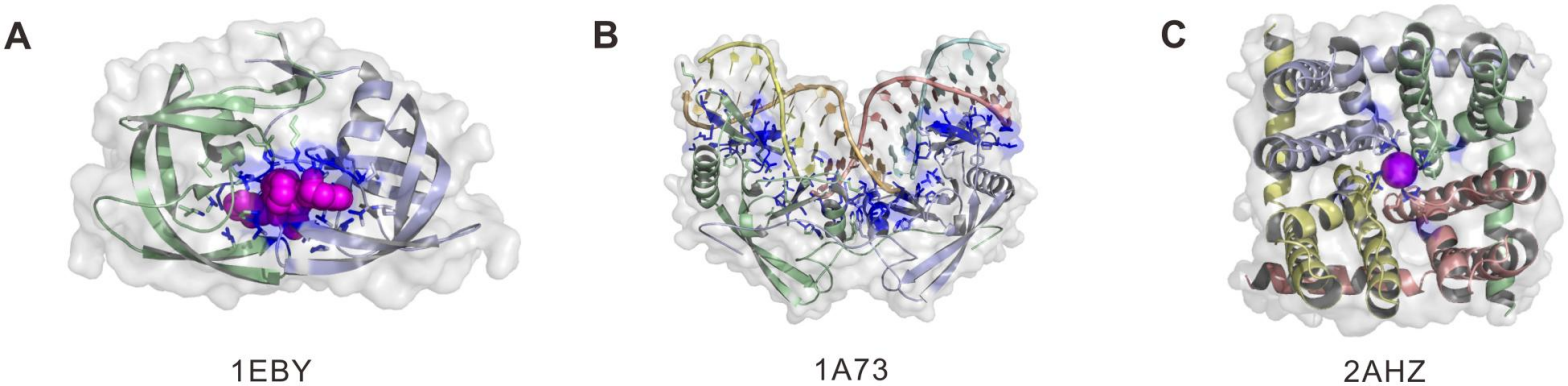
Three examples of protein–ligand interactions in Q-BioLiP **A**. The interaction between the HIV-1 protease and its inhibitor (PDB: 1EBY). **B**. The DNA ligand binding with the structure of an intron-encoded endonuclease (PDB: 1A73). **C**. The structure of the tetrameric NaK channel binding with K^+^ (PDB: 2AHZ). HIV-1, human immunodeficiency virus-1.

One of the key developments of Q-BioLiP over BioLiP is the change in the form of protein structure. Q-BioLiP improves the representation of protein structure from single chain-based tertiary structure to quaternary structure. As indicated above, the quaternary structure may consist of single chain or multiple chains. The completeness of the protein structure can ensure the completeness of ligand-binding interactions [18]. For instance, **Figure 2**A shows the interaction between the human immunodeficiency virus-1 (HIV-1) protease and its inhibitor (PDB ID: 1EBY), where the protease is a C2 symmetric homo dimer and the catalytic residues are located on the interface. This interaction was however separated into two individual entries in BioLiP (each for one receptor chain) (Figure S1).

#### State of DNA/RNA chains

With the pairing algorithm introduced above, we are able to correctly pair DNA/RNA chains. For example, the DNA ligand binding with the structure of an intron-encoded endonuclease is formed by four DNA chains (PDB ID: 1A73) (Figure 2B). On the contrary, BioLiP separates the interactions into 6 individual entries (Figure S2): 2 receptor chains, and 3 DNA chains binding with each receptor chain (one DNA chain is ignored as it does not interact with the receptor chain after separation).

#### Binding affinity

In Q-BioLiP, ∼ 49,000 entries are annotated with experimental binding affinity data. In addition, predicted binding affinity and binding interface area are also provided for ∼ 1.7 million protein–small molecule interactions (peptide and DNA/RNA ligands are excluded).

#### Data category

Only interactions judged as biologically relevant are kept in BioLiP. However, this may result in the omission of some biologically relevant interactions due to misjudgement. For example, the structure of the tetrameric NaK channel binding with K^+^ (PDB ID: 2AHZ) was judged as biologically irrelevant in BioLiP. In fact, the ion K^+^ is located in the pocket of a tetrameric cation channel and thus biologically relevant. This is correctly captured in Q-BioLiP due to the utilization of quaternary structure (Figure 2C). Around 64,000 Q-BioLiP entries (involving 12,000 PDB entries), initially filtered out by BioLiP, have been retained in Q-BioLiP due to the consideration of quaternary structure, and rule adjustments.

We admit that it is also possible that the judgement of biological relevance in Q-BioLiP is not perfect. We thus keep all data in our database so that the users can decide the relevance based on their own expertise. For the sake of completeness, structures with ligands or proteins only are also stored in our database, which may be used for other purposes, such as in protein structure database searching. To summarize, the Q-BioLiP data are organized into three categories: biologically relevant interactions; biologically irrelevant interactions; and structures with ligands or proteins only.

### Statistics of the Q-BioLiP data

**Figure 3:**
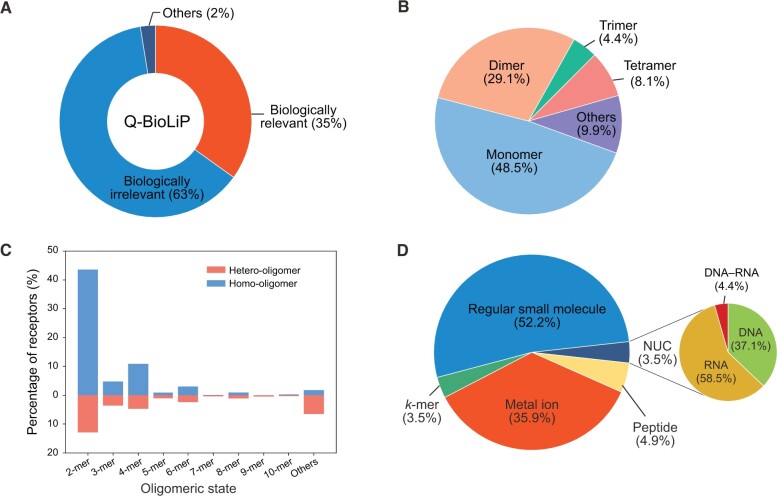
Statistics of the Q-BioLiP data **A**. Overall statistics for the Q-BioLiP entries. **B**. Distribution of receptors in terms of the oligomeric state. **C**. Distribution of receptors in terms of homo-oligomers and hetero-oligomers at different oligomeric states. **D**. Distribution of ligands. Note that the data shown in panels B–D only involve the biologically relevant interactions. NUC, nucleic acid ligand.

By the time of October 11th, 2023, Q-BioLiP contains about 2.7 million entries, involving about 210,000 structures from PDB. These structures are determined by the following experimental methods (Figure S3): X-radiation (X-ray) (85%), NMR (6.7%), cryo-EM (8.2%), and others (1%). The overall distribution of the Q-BioLiP entries is shown in **Figure 3**A. Among the total entries, 35% and 63% are annotated as biologically relevant interactions and biologically irrelevant interactions, respectively; the remaining 2% are structures with ligands or proteins only. Further analysis was conducted on the biologically relevant interactions, which are the core data of Q-BioLiP.

The distribution of the proteins involved in biologically relevant interactions is shown in Figure 3B and C. Figure 3B shows that almost half (48.5%) of these proteins are monomers, while the remaining half contain more than one chains (called oligomers). Interestingly, oligomers with even numbers of chains (*e.g.*, dimers and tetramers) are more than those with odd numbers of chains (*e.g.*, trimers). The oligomers can be divided into two groups, homo-oligomers and hetero-oligomers, depending on if all chains are identical or not. Figure 3C shows the proportion of homo-oligomers and hetero-oligomers at different oligomeric states. For oligomers with even numbers of chains (especially for dimers and tetramers), the number of homo-oligomers tends to be higher than the number of hetero-oligomers.

Figure 3D shows the proportion of different ligand types involved in biologically relevant interactions. Regular small molecules account for more than half (52.2%) of all ligands; metal ions are the second largest group (35.9%). For nucleic acid ligands (3.5%), 58.5% and 37.1% of them are RNA and DNA, respectively; the remaining 4.4% are DNA–RNA complex.

Among the biologically relevant interactions involving oligomeric targets, 28.8% of the ligands interact with more than one chains (Figure S4A), which spreads across states from dimers to 10-mers (Figure S4B). This indicates the necessity of updating BioLiP to Q-BioLiP, to maintain the completeness of protein–ligand interactions.

### Derivation of interaction-based data from Q-BioLiP

Besides protein–small molecule interaction, a variety of interaction data are derived from Q-BioLiP, which are shown in **Table 2**. These data can be easily used to construct training and/or benchmark datasets in the development of methods for the modelling of protein–small molecule interactions, protein–metal ion interactions [26], protein–peptide interactions [27], protein–protein interactions [28], protein–DNA/RNA interactions [29], and RNA–small molecule interactions [30]. These data are available for download at the Download page.

**Table 2:** Interaction-based data derived from Q-BioLiP

Interaction type	No. of redundant interactions (No. of receptors)	No. of non-redundant interactions (No. of receptors)
Protein–small molecule	∼ 460,000 (∼ 140,000)	∼ 110,000 (∼ 28,000)
Protein–metal ion	∼ 300,000 (∼ 79,000)	∼ 73,000 (∼ 19,000)
Protein–peptide	∼ 31,000 (∼ 20,000)	∼ 8000 (∼ 4000)
Protein–protein	∼ 150,000 (N/A)	∼ 38,000 (N/A)
Protein–DNA	∼ 10,000 (∼ 8000)	∼ 3000 (∼ 2000)
Protein–RNA	∼ 12,000 (∼ 6000)	∼ 4000 (∼ 2000)
RNA–small molecule	∼ 21,000 (∼ 3000)	∼ 4000 (∼ 200)

*Note*: The numbers in parentheses denote the numbers of receptors (*i.e.,* the protein structures or RNA structures); protein–protein refers to protein oligomer structures (*i.e.,* with ≥ 2 chains).

### Binding affinity data

About 49,000 entries in Q-BioLiP (from ∼ 21,000 unique PDB entries) are annotated with experimental binding affinities. Predicted binding affinities are provided for other entries without experimental binding affinities. Benchmark test on 285 high-quality data from the PDBbind core set (v2016) showed that the binding affinities predicted by these tools correlated well with the experimental binding affinities, with Pearson’s correlation coefficient (PCC) around 0.6 (Figure S5A–C). The consensus binding affinity prediction based on a regression model yielded a 6.3%–17.2% higher PCC than the individual predictions (Figure S5D). Further tests based on a bootstrap sampling on the dataset by 1000 times showed that the improvement was statistically significant (Figure S6).

## Database interface

**Figure 4:**
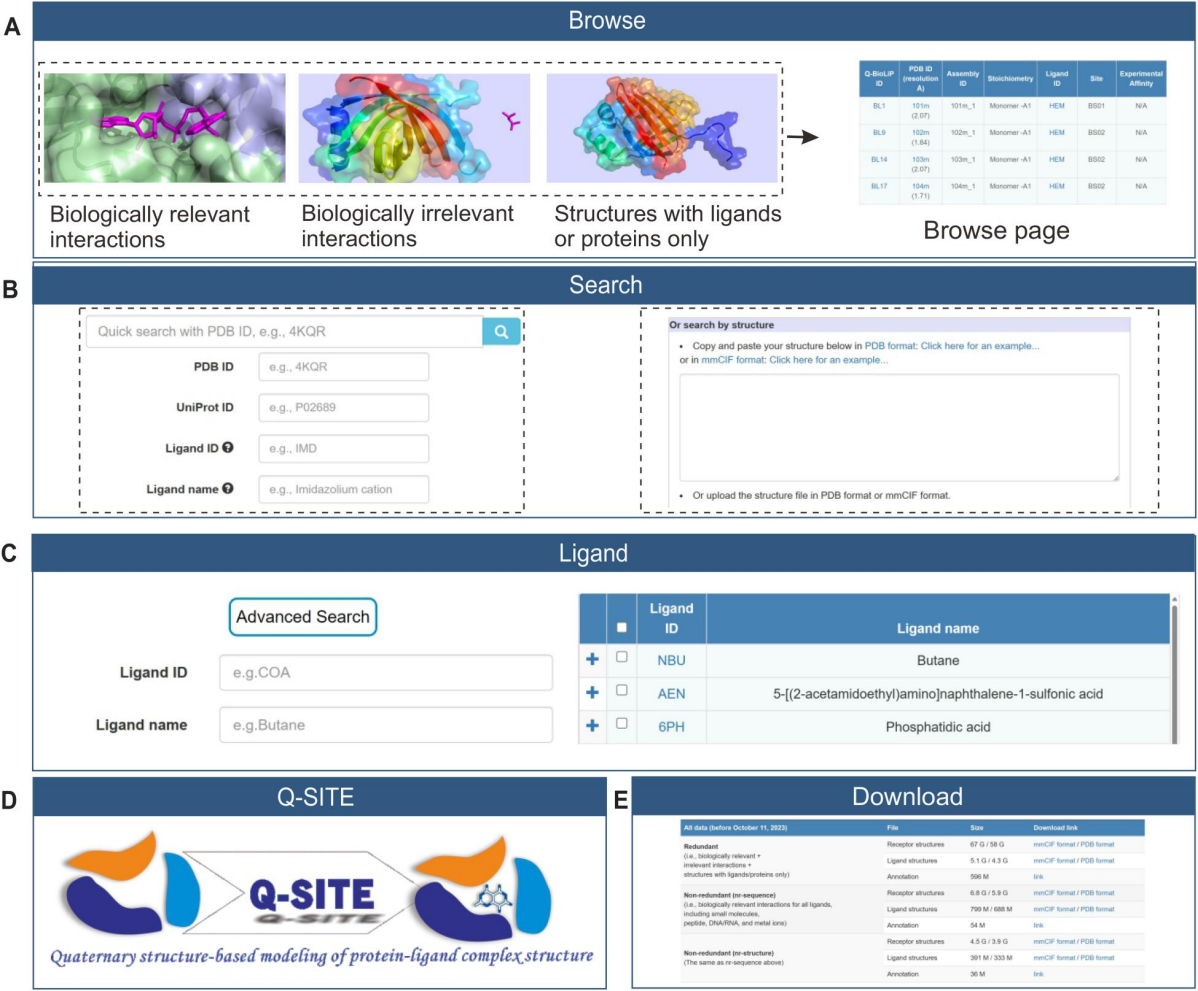
Major modules of the Q-BioLiP web interface **A**. Through the Browse module, users can browse entries of three categories. **B**. Through the Search module, users can perform search with two options. **C**. Through the Ligand module, users can search for detailed information of a specific ligand or browse all ligands. **D**. A quaternary structure-based protein–ligand complex modelling module Q-SITE is provided, which supports structure inputs with both monomers and oligomers. **E**. Through the Download page, all data in Q-BioLiP can be downloaded freely.

The Q-BioLiP database is freely accessible at https://yanglab.qd.sdu.edu.cn/Q-BioLiP/. Five modules are provided to use the data (**Figure 4**), which are introduced below.

### Browse module

There are three options in the Browse module: (1) Browse biologically relevant entries, (2) Browse biologically irrelevant entries, and (3) Browse structures with ligands or proteins only (Figure 4A). Clicking on each option would display the summary of entries in the corresponding category in the form of a table, which has seven columns: Q-BioLiP ID, PDB ID with resolution, assembly ID, stoichiometry, ligand ID, site, and experimental affinity. The detailed information for each entry is available by clicking on the corresponding Q-BioLiP ID.

### Search module

In the Search module, users can query the Q-BioLiP data using two options. The first one is a rapid search of the following items: PDB ID, UniProt ID, ligand ID, and ligand name. An input box is provided to filter results further. The results can be downloaded in several formats including JSON and CSV. Another one is protein structure-based search using the Foldseek algorithm [31], which usually takes ∼ 2 min to complete.

### Ligand module

In this module, all ligands involved in Q-BioLiP are displayed in two columns by default: ligand ID and ligand name. More detailed information can be shown by clicking on the ligand ID link. The detailed ligand page contains the 2-dimensional (2D) view of a ligand and its synonyms, as well as its chemical component summary. To sufficiently annotate ligands, the synonyms for each ligand were collected from PDB [20], Chemical Entities of Biological Interest (ChEBI) [32], and PubChem [33], which are also essential in the biological relevance assessment. The chemical component summary includes molecular weight, chemical formula, the simplified molecular input line entry system (SMILES) string, and external links. Besides, a search module (the “Advanced Search” button) is provided for searching for a specific ligand by ligand ID or ligand name.

### Q-SITE module

Q-SITE is an extension of the COACH-D algorithm [6] for template-based modelling of protein–ligand complex structures. Q-SITE supports the structure inputs with both oligomers and monomers. The modelling is based on quaternary structure templates in Q-BioLiP, which is about 40 times faster and 6.5% more accurate than COACH-D. More details about Q-SITE will be introduced elsewhere. Users can provide either experimental structure or predicted structure as inputs, and the server will return the top 5 predictions within ∼ 20 min on average.

### Download module

The Q-BioLiP data are provided for download at this module. Three tables are provided on the Download page: All data, Interaction-based data, and Weekly update. In the “All data” table, both redundant and non-redundant versions are provided for easy download. The redundant version contains ∼ 2.6 million entries of interactions (*i.e.*, biologically relevant interactions + biologically irrelevant interactions) and ∼ 68,000 entries with proteins or ligands only.

For the core data of the biologically relevant interactions, two non-redundant datasets (denoted by nr-sequence and nr-structure) were created based on sequence similarity and structure similarity, respectively. As quaternary structure may contain multiple chains, we defined redundancy as follows. First, the protein structures were grouped by the oligomeric state (*i.e.,* the number of chains). Second, the chain-level sequences and structures were clustered by cd-hit [34] and Foldseek [31], respectively. To identify redundant structures within each group, we compared pairs of chains from the two structures. If any pair of chains exhibited a similarity (*i.e.,* sequence identity or Foldseek score) higher than 0.9, those structures were considered redundant. The nr-sequence dataset has ∼ 41,000 receptors and ∼ 240,000 ligands, whereas the nr-structure dataset has ∼ 29,000 receptors and ∼ 160,000 ligands. This suggests that protein structures are more conserved than protein sequences. Analysis shows that the distributions of the ligands binding to multiple chains or single chain are similar to the redundant dataset (Figure S4 C–F).

Within the “Interaction-based data” table, users can download the previously mentioned interaction-based datasets. Given that some users may be unfamiliar with the mmCIF format, we provide a script to convert between mmCIF format and PDB format. This script is designed to automatically detect the input format and convert it to the other format. Q-BioLiP is updated weekly, following the PDB update. The updated data are provided for download in the “Weekly update” table, to avoid re-download of the previous data.

For users who are interested in monomer structure-based protein–ligand interactions, we also provide the redundant data in single-chain format like BioLiP. They were obtained by splitting the quaternary structures into individual receptor chains. The data are available for download at a separate link (https://yanglab.qd.sdu.edu.cn/Q-BioLiP/Download/index_biolip.html).

### Detailed information on protein–ligand interactions

**Figure 5:**
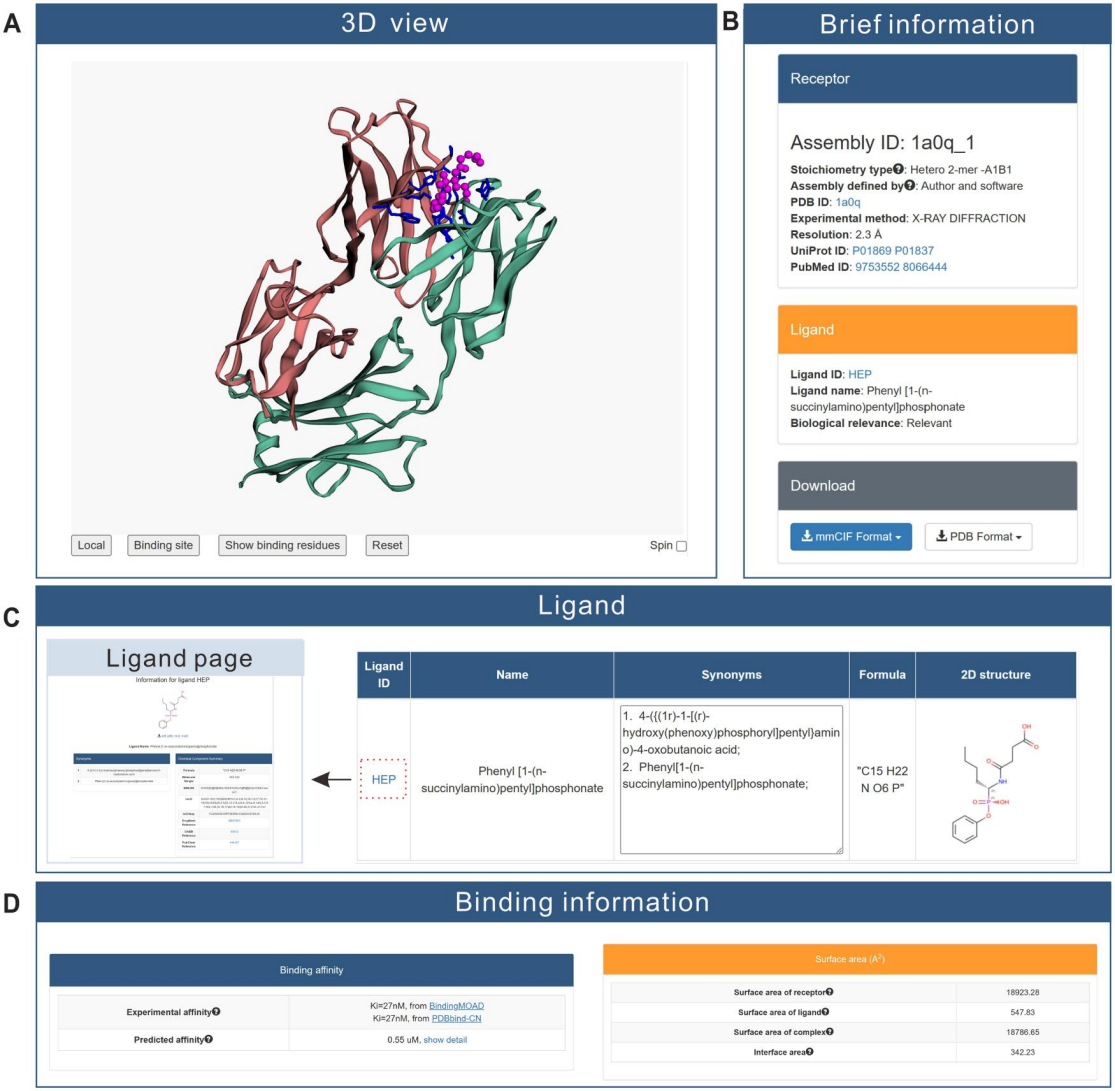
Information displayed on the webpage for an example Q-BioLiP entry **A**. The 3D visualization of the protein–ligand interaction. **B**. The overall brief information about Receptor, Ligand, and Download. **C**. The detailed ligand information, including ligand ID, ligand name, synonyms, formula, and 2D structure. **D**. The binding affinity and surface area. 3D, 3-dimensional; 2D, 2-dimensional.

The information on protein–ligand interactions is presented as a webpage, which consists of three tabs. The first tab is the 3D visualization of the interaction (**Figure 5**A). The visualization is powered by the 3Dmol.js package [35]. Two buttons are provided to show the local view and specific binding residues, respectively. In addition, the specific binding residues can be shown in a table by clicking on the “Show binding residues” button. On the right of the page (Figure 5B), the overall information is organized into three sections: Receptor, Ligand, and Download. In the Receptor section, the stoichiometry type is derived with the same definition as in PDB, *i.e.*, two chains are considered equivalent if their sequence identity is greater than 95%. In the Ligand section, three items are shown: ligand ID, ligand name, and biological relevance. In the Download section, the structures of receptor and ligand are provided in both mmCIF and PDB formats. Structures comprising more than 62 chains are currently unavailable in the legacy PDB format. In order to maintain completeness, these large structures are divided into multiple PDB files. A tarball (.tar.gz) is provided on the page, which is a bundle of these files along with a text file to map the original chain IDs to the new IDs.

The second tab is the ligand information (Figure 5C), including ligand ID (with a link to the detailed information page), ligand name, synonyms, formula, and 2D image of the ligand structure.

The last tab is the binding information (Figure 5D), which consists of the binding affinity and the surface area. Both experimental (if available) and predicted binding affinities are provided.

## Conclusion

We have developed the Q-BioLiP database for quaternary structure-based protein–ligand interactions. The major contributions include: (1) all structures in Q-BioLiP are based on quaternary structures, making the protein–ligand interactions complete; (2) DNA/RNA chains are properly paired; (3) both experimental and predicted binding affinities are provided; (4) to address the problem of misjudgement of biological relevance, both relevant and irrelevant entries are kept; and (5) a quaternary structure-based protein–ligand complex modelling algorithm is developed. We believe that the development of Q-BioLiP will better serve the community of protein–ligand interactions.

During the submission of this work at the beginning of July 2023, the authors noted an updated version of the original BioLiP (*i.e.*, BioLiP2 [36], https://zhanggroup.org/BioLiP). Q-BioLiP and BioLiP2 were developed independently based on the original BioLiP database. They are complementary to each other in terms of both web interface and underlying data. Our Q-BioLiP focuses on improving the quality of protein–ligand interaction data, as summarized above; while BioLiP2 aims to improve the usability of the database. Therefore, we believe that both databases are valuable to the community of protein–ligand interactions.

## Supplementary Material

qzae001_Supplementary_Data

## Data Availability

Q-BioLiP is freely available at https://yanglab.qd.sdu.edu.cn/Q-BioLiP/.

## CRediT author statement

**Hong Wei:** Data curation, Software, Writing – original draft, Writing – review & editing. **Wenkai Wang:** Writing – original draft. **Zhenling Peng:** Supervision, Writing – original draft. **Jianyi Yang:** Conceptualization, Methodology, Supervision, Writing – review & editing. All authors have read and approved the final manuscript.
