# Supplementary material for: Q-BioLiP: A Comprehensive Resource for Quaternary Structure-based Protein–ligand Interactions
Source: Genomics Proteomics Bioinformatics. 2024 Jan 4;22(1):qzae001. doi: 10.1093/gpbjnl/qzae001 (PMC11423850; doi:10.1093/gpbjnl/qzae001)
Supplement: qzae001_Supplementary_Data [file qzae001_supplementary_data.zip › Supplementary figure caption.docx]

**Supplementary material**

**Figure S1** **The difference between Q-BioLiP and BioLiP for an example structure (PDB ID: 1EBY)**

**A.** The chain A of the HIV-1 protease bound with its inhibitor (BioLiP entry). **B.** The chain B of the HIV-1 protease bound with its inhibitor (BioLiP entry). **C.** The complete structure HIV-1 protease bound with its inhibitor (Q-BioLiP entry).

**Figure S2 The difference between Q-BioLiP and BioLiP for an example protein–DNA interaction (PDB ID: 1A73)**

The DNA chains C (**A**), E (**B**), and F (**C**) bound with the protein chain A, respectively (BioLiP entry). The DNA chains C (**D**), E (**E**), and D (**F**) bound with the protein chain B, respectively (BioLiP entry). **G.** The complete DNA structure bound with the complete protein structure in Q-BioLiP.

**Figure S3 Distribution of structure determination methods**

The proportion of structures in PDB (**A**), biologically relevant entries (**B**), biologically irrelevant entries (**C**), and other entries (**D**). Others structure determination methods include EPR, neutron diffraction, and so on. NMR, nuclear magnetic resonance; X-ray, X-radiation; cryo-EM, cryo-electron microscopy; EPR, electron paramagnetic resonance.

**Figure S4 Distributions of the ligand binding data**

The proportion of ligand binding with single chain and multiple chains in the redundant (**A**), nr-sequence (**C**), nr-structure datasets (**E**), respectively. The distribution of ligand binding with single chain and multiple chains in redundant (**B**), nr-sequence (**D**), nr-structure datasets (**F**) at different oligomeric states, respectively. nr-sequence, non-redundant datasets were created based on sequence similarity; nr-structure, non-redundant datasets were created based on structure similarity.

**Figure S5 Relationship between the predicted and the real binding affinities**

The relationship between the real affinity and ITScore (**A**), AutoDock Vina (**B**), and X-Score (**C**), respectively. **D.** The relationship between the real affinity and the consensus method. PCC, Pearson’s correlation coefficient.

**Figure S6 Distributions of the PCCs between predicted and real binding affinities by different methods**

The distributions were obtained based on bootstrap sampling (1000 times).
