# Supplementary figures and images for: Q-BioLiP: A Comprehensive Resource for Quaternary Structure-based Protein–ligand Interactions
Source: Genomics Proteomics Bioinformatics. 2024 Jan 4;22(1):qzae001. doi: 10.1093/gpbjnl/qzae001 (PMC11423850; doi:10.1093/gpbjnl/qzae001)

**A**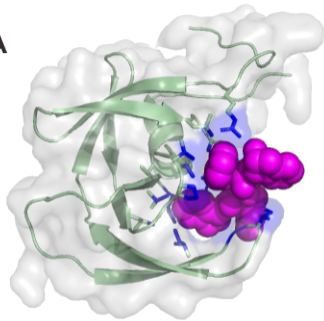

1EBY (A)

**B**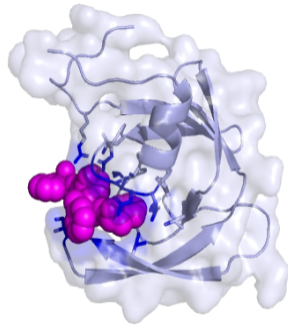

1EBY (B)

**C**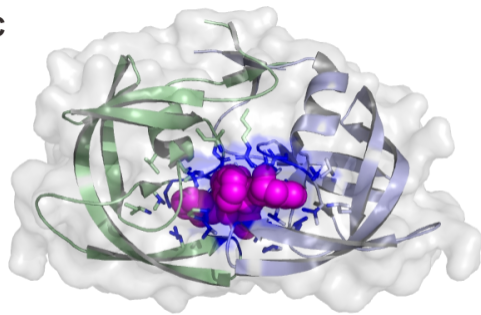

1EBY (A-B)

Supplement: qzae001_Supplementary_Data [file qzae001_supplementary_data.zip › FigureS1.pdf]

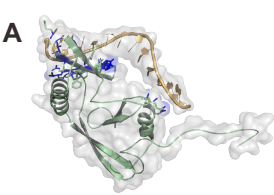

1A73 (A:C)

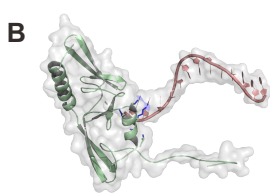

1A73 (A:E)

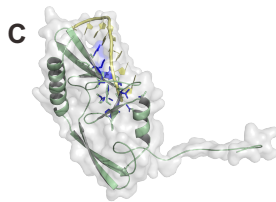

1A73 (A:F)

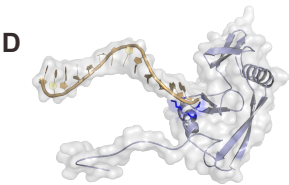

1A73 (B:C)

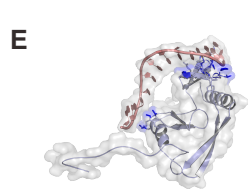

1A73 (B:E)

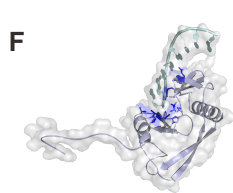

1A73 (B:D)

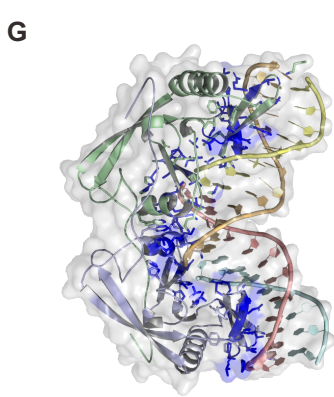

1A73 (A-B: C-D-E-F)

Supplement: qzae001_Supplementary_Data [file qzae001_supplementary_data.zip › FigureS2.pdf]

**A**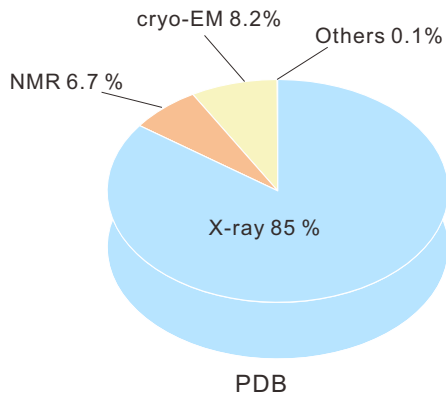**B**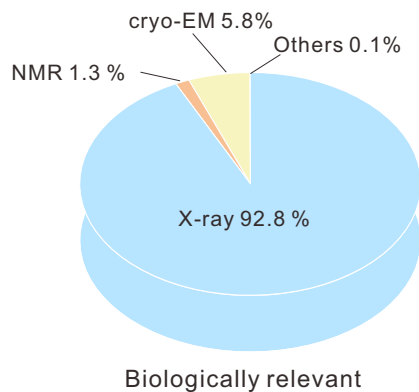**C**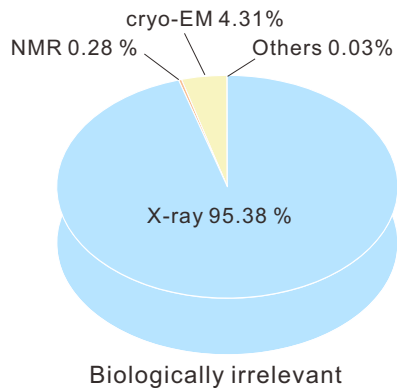**D**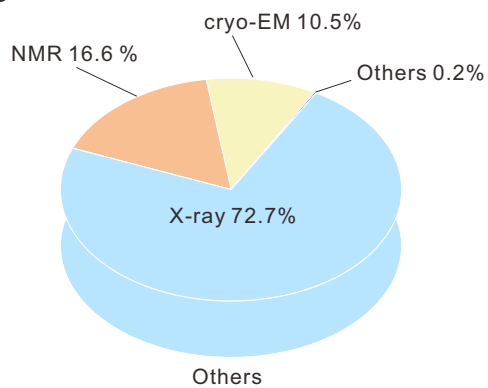

Supplement: qzae001_Supplementary_Data [file qzae001_supplementary_data.zip › FigureS3.pdf]

**A**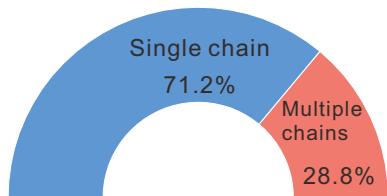

Redundant

**C**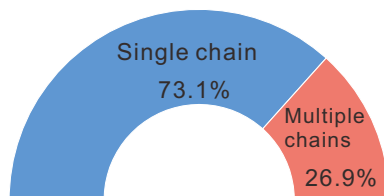

nr-sequence

**E**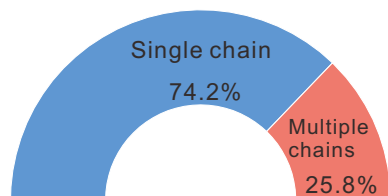

nr-structure

**B**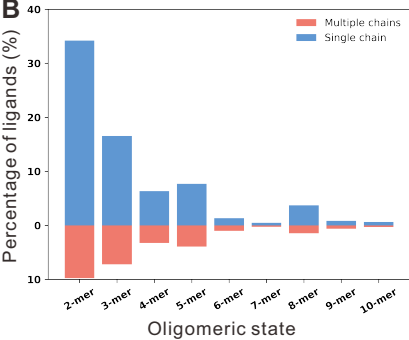**D**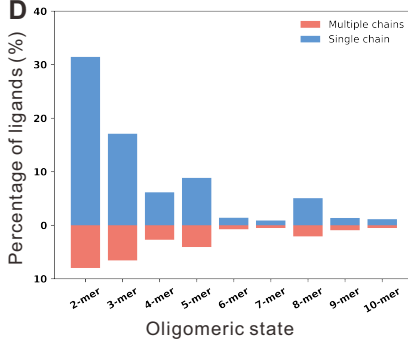**F**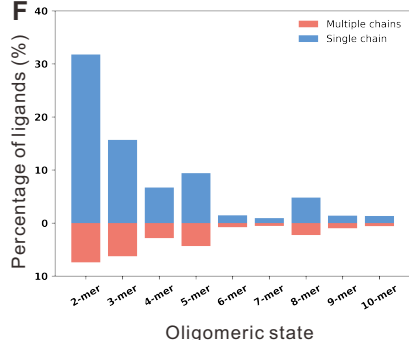

Supplement: qzae001_Supplementary_Data [file qzae001_supplementary_data.zip › FigureS4.pdf]

**A**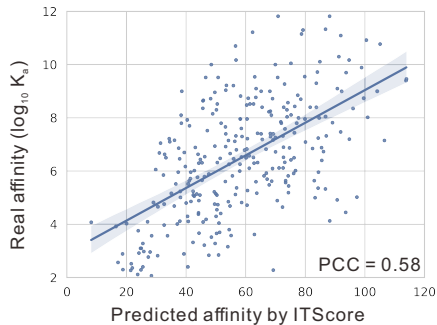**B**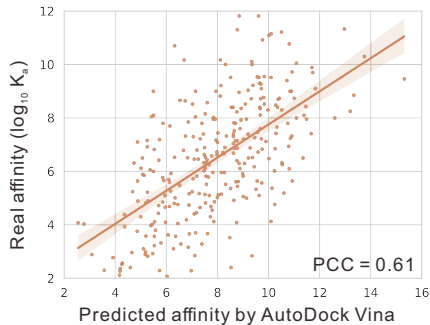**C**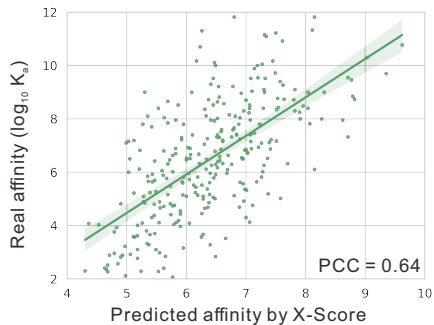**D**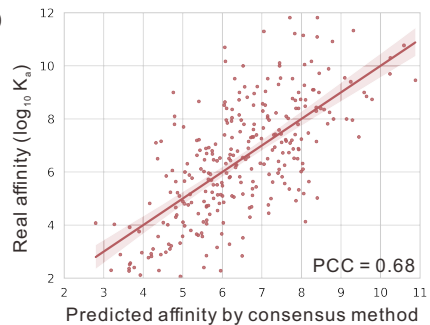

Supplement: qzae001_Supplementary_Data [file qzae001_supplementary_data.zip › FigureS5.pdf]

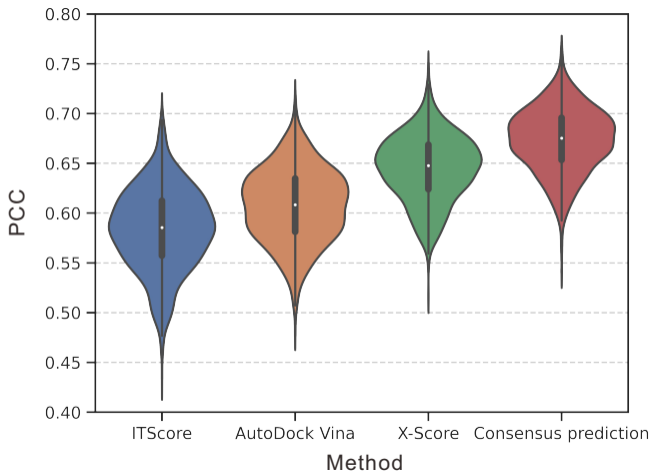

Supplement: qzae001_Supplementary_Data [file qzae001_supplementary_data.zip › FigureS6.pdf]
